# Modeling Uremic Vasculopathy With Induced Pluripotent Stem Cell-Derived Endothelial Cells as a Drug Screening System

**DOI:** 10.3389/fcell.2020.618796

**Published:** 2021-01-12

**Authors:** Hye Ryoun Jang, Hyung Joon Cho, Yang Zhou, Ning-Yi Shao, Kyungho Lee, Hoai Huong Thi Le, Junseok Jeon, Jung Eun Lee, Wooseong Huh, Sang-Ging Ong, Won Hee Lee, Yoon-Goo Kim

**Affiliations:** ^1^Division of Nephrology, Department of Medicine, Samsung Medical Center, Stem Cell & Regenerative Medicine Institute(SCRMI), Sungkyunkwan University School of Medicine, Seoul, South Korea; ^2^School for Engineering of Matter, Transport & Energy, Arizona State University, Tempe, AZ, United States; ^3^Stanford Cardiovascular Institute, Stanford University School of Medicine, Stanford, CA, United States; ^4^Health Sciences, University of Macau, Macau, China; ^5^Department of Basic Medical Sciences, University of Arizona College of Medicine, Phoenix, AZ, United States; ^6^Department of Pharmacology & Regenerative Medicine, University of Illinois College of Medicine, Chicago, IL, United States; ^7^Division of Cardiology, Department of Medicine, University of Illinois College of Medicine, Chicago, IL, United States

**Keywords:** endothelial cells, induced pluripotent stem cells, uremic vasculopathy, uremic toxin, chronic kidney disease

## Abstract

**Background:** Cardiovascular complications are the leading cause of mortality in patients with chronic kidney disease (CKD). Uremic vasculopathy plays a crucial role in facilitating the progression of cardiovascular complications in advanced CKD. However, the improvement of conventional research methods could provide further insights into CKD.

**Objectives:** In this study, we aimed to develop a novel model of uremic vasculopathy as a potential drug screening system.

**Methods and Results:** The effects of uremic serum and different combinations of uremic toxins on induced pluripotent stem cell (iPSC)-derived endothelial cells (ECs) of a normal control and a CKD patient were investigated using several functional assays. We found that a mixture of uremic toxins composed of high urea, creatinine, uric acid, and indoxyl sulfate exerted deleterious effects on normal control iPSC-ECs that were comparable to uremic serum by increasing reactive oxygen species and apoptosis, as well as suppression of tube formation. Additional characterization revealed a potential involvement of dysregulated TGF-β signaling as treatment with either losartan or TGF-β inhibitors led to the attenuation of adverse effects induced by uremic toxins. Importantly, impaired wound healing potential seen in CKD patient-specific iPSC-ECs was rescued by treatment with losartan and TGF-β inhibitors.

**Conclusion:** Our study demonstrated that simplified uremic toxin mixtures can simulate the uremic micromilieu reproducibly and CKD patient-specific iPSC-ECs can potentially recapitulate susceptibility to uremic vasculopathy. This novel model of uremic vasculopathy may provide a new research tool as a drug screening system.

## Introduction

Cardiovascular complications are the leading cause of mortality and major morbidities in patients with chronic kidney disease (CKD) (Go et al., 2004; Tonelli et al., 2006; Stenvinkel, 2010). Uremic vasculopathy is the cornerstone of cardiovascular complications acting as the primary factor that accelerates atherosclerosis and heart failure (Levin et al., 1990; Deppisch et al., 2001; Jourde-Chiche et al., 2011; Sallée et al., 2014). It also remains an important hurdle for kidney transplantation in end-stage renal disease (ESRD) patients undergoing longstanding dialysis by causing severe atherosclerosis and heavy vascular calcification (Jourde-Chiche et al., 2011; Torres and de Broe, 2012). Although epidemiologic research can provide insightful results from CKD patients in the real world, large sample sizes with very long follow-up periods are required and the results can often be confounded by factors such as comorbidities other than CKD (Ballew and Matsushita, 2018). Therefore, studies revealing the pathophysiology of uremic vasculopathy and investigation of potential new drugs or the adequacy of current drugs are required to improve overall outcomes for CKD patients.

Previous studies using genetically engineered or surgically simulated murine CKD models have provided significant insights into the pathophysiology of uremia-induced cardiovascular complications (Baraka and El Ghotny, 2012; Xie et al., 2015). However, these murine models have limitations for investigating the effects of uremia on vessels because of interspecies differences and methodological limitations in simulating CKD or evaluating changes in vessels. To investigate the pathophysiology of uremic vasculopathy thoroughly in the hope of identifying new drugs, a novel model of uremic vasculopathy with human endothelial cells (ECs) that reflects genetic susceptibility to uremic toxins is essential.

In this study, we developed a novel model of uremic vasculopathy using ECs differentiated from human induced pluripotent stem cells (iPSCs). The effects of several uremic toxins on iPSC-ECs were compared with those of uremic serum from ESRD patients. The feasibility of this new model as a novel drug screening tool was also investigated.

## Materials and Methods

### Study Participants and Design

Serum and peripheral blood mononuclear cells (PBMCs) were collected from 5 healthy volunteers and 5 ESRD patients receiving hemodialysis for more than a year. Informed consent was obtained from all subjects. The Institutional Review Board of the Samsung Medical Center approved the study protocol in compliance with the Declaration of Helsinki (October 2008), and informed consent was waived because of the retrospective and non-interventional design of the study (IRB number: 2016-11-025).

Several functional assays for comparing the effects of serum from normal controls and ESRD patients as well as simplified uremic toxin mixtures on iPSC-ECs were performed for establishing a new model of uremic vasculopathy.

The wound healing potential of normal control iPSC-ECs and ESRD patient-specific iPSC-ECs was also compared by a scratch migration assay.

### Generation of iPSC-ECs

PBMCs from a normal control and an ESRD patient were reprogrammed into iPSCs using Sendai virus (Takahashi et al., 2007; Churko et al., 2013). ECs were differentiated from iPSCs according to the protocol validated in previous studies (Sa et al., 2017; Bezenah et al., 2018; Lee et al., 2019). Briefly, the iPSCs were treated with a differentiation induction medium (RPMI and B-27 minus insulin, Thermo Fisher Sci, Waltham, MA) supplemented with 6 μM and 2 μM of glycogen synthase kinase 3-β inhibitor, CHIR-99021 (Selleck Chemicals, Houston, TX), on day 0 and day 2, respectively. From day 4 to 12 of differentiation, cells were cultured in different combinations of differentiation induction media and EGM-2 media from Lonza (100% differentiation medium on day 4, 50% differentiation and 50% EGM-2 media on day 6, 25% differentiation and 75% EGM2 media on day 8, and 100% EGM2 medium on day 10) with growth factors including 50 ng/ml VEGF, 20 ng/ml FGF2, and 20 ng/ml BMP4 (PeproTech, Rocky Hill, NJ). On day 12 post-differentiation, cells were sorted by a magnetic cell sorting (MACS) system (Miltenyi Biotech, San Diego, CA) using magnetic beads conjugated to human CD144 antibodies, as directed by the manufacturer, and expanded on 0.2% gelatin-coated plates. iPSC-ECs were then cultured in EGM2 medium at 37°C and 5% CO_2_ in a humidified incubator with medium changes every other day. The experiments described in this manuscript were performed between passages 2–4. The phenotypes of iPSC-ECs used in this study were characterized in previous studies (Lee et al., 2019; Ong et al., 2019).

### Serum Sample Preparation and Analytic Procedure

For serum preparation, blood was separated by centrifuging clotted blood at 3,500 rpm for 15 min at 4°C and was aliquoted before storing at −80°C until needed for experiments. Serum uric acid, creatinine, and blood urea nitrogen were measured using Fuji Dri-Chem 7000i (Fujifilm Corporation, Tokyo, Japan).

### Preparation of Uremic Toxin Mixtures

Urea, creatinine, indoxyl sulfate (Sigma-Aldrich, Louis, MO), and advanced glycation end-products (AGEs, BioVision, San Francisco, CA) were dissolved in Dulbecco's phosphate-buffered saline and uric acid (Sigma-Aldrich) was dissolved in 1 M NaOH, then the solutions were filtered (0.22 μm pore size) before experiments.

### Luminex Multiplex Assay

Serum samples were mixed with antibody-linked polystyrene beads on 96-well filter plates and incubated at room temperature for 2 h, followed by overnight incubation at 4°C on an orbital shaker at 500–600 rpm. After washing plates twice with wash buffer, a biotinylated detection antibody was added for 2 h at room temperature with shaking. Samples were then filtered, washed twice, and resuspended in streptavidin-PE for 40 min at room temperature. Two additional vacuum washes were performed before adding the reading buffer. All standards and samples were measured in duplicate. Plates were read using a Luminex 200 instrument with a lower bound of 100 beads per sample per cytokine. For quality control, custom assay control beads (Radix Biosolutions, Georgetown, TX) were added to all wells.

### Assays for Cellular Viability, Reactive Oxygen Species (ROS), and Caspase 3/7 Activity

iPSC-ECs plated on 96-well plates were subjected to plate-based assays after serum (15%) or uremic toxin mixture treatments. Assays using CellTiter-Glo 2.0, ROS-Glo H_2_O_2_, and Caspase-Glo 3/7 (Promega, Madison, WI) were performed following the manufacturer's instruction and the luminescence signal was recorded on the GloMax-Multi detection system (Promega). Cells in the original sample plate were kept for measuring the total cell number by calcein-AM (Thermo Fisher Scientific) to allow normalization.

### Measurement of Endothelial Function

An *in vitro* endothelial tube formation assay was carried out following the manufacturer's instructions. Briefly, after coating the 15-well μ-Slice Angiogenesis (Ibidi GmbH, Gräfelfing, Germany) with Corning Matrigel Basement Membrane Matrix, iPSC-ECs pre-treated with serum or uremic toxin mixture (UT)-H with or without drugs for 48 h were seeded at 1 × 10^4^ cells/well. After 16 h incubation, capillary network images were taken using a Revolve microscope and quantitation was made using ImageJ.

For the *in vitro* migration assay, normal control iPSC-ECs and ESRD patient-specific iPSC-ECs were treated with drugs for 48 h before seeding in 24-well plate (5 × 10^4^ cells/well). When cells reached 80% confluence, the culture medium was replaced overnight for serum starvation. A straight line was scraped in the cell monolayer with a sterile 200 μl pipette tips and the debris was removed by washing twice with media. Cells were then incubated with EGM2 media and imaged at 0, 4, and 10 h after the scratch. The area and width of the scratch were analyzed with ImageJ. Losartan (a well-known angiotensin-receptor blocker; Sigma), SD-208 (a selective TGF-β receptor I kinase (ALK5) inhibitor; Selleckchem, Houston, TX), and LY2109761 (TGF-β receptor I and type II dual inhibitor; Selleckchem) were used for drug treatment.

### Human TGF-β Pathway Protein Phosphorylation

After treating iPSC-ECs with either the control or UT-H for 24 h, the relative levels of phosphorylation of 8 TGF-β pathway proteins in cell lysates were quantified using human TGF-β pathway phosphorylation array (C1 series, RayBiotech, Peachtree Corners, GA) following the manufacturer's instructions. Briefly, after blocking each well, samples were incubated for 2 h at room temperature with membranes arrayed with antibodies against 8 TGF-β phosphorylated proteins. After washing twice in wash buffer, a detection antibody cocktail was added into each well for 2 h at room temperature. After washing, membranes were then incubated with horseradish peroxidase (HRP)-rabbit IgG for 2 h, washed, and placed in the chemiluminescence detection buffer. FluorChem E (Protein Simple, San Jose, CA) was used to detect signal chemiluminescence intensities from protein array membranes. Quantitative analysis for chemiluminescent intensity was conducted using Image J.

### RNA-seq Library Preparation and Analysis

Total RNA was first extracted from iPSC-ECs using a RNeasy Mini Kit (Qiagen, Hilden, Germany) according to the manufacturer's instruction and 100 ng RNA was used to construct sequencing libraries. Libraries were prepared using the TruSeq Stranded Total RNA Library Prep Kit (Illumina, San Diego, CA) and the raw 150 bp paired-end RNA-seq reads sequenced by Illumina HiSeq 2000 were trimmed by TrimGalore version 0.4.2 to exclude adapter sequences and bases with Phred scores of <20, providing a low probability of base-calling error (*p* < 1%) equivalent to >99% accuracy. The reads were aligned to the human genome hg38 by Hisat2 (Pertea et al., 2016), and annotated by FeatureCounts of the Subread package (Liao et al., 2014). Differentially expressed genes were detected by DESeq2 (Love et al., 2014). The gene ontology enrichment analyses were implemented by Metascape (Zhou et al., 2019).

### Statistical Analyses

Data are expressed as the mean ± standard error of the mean (SEM) or mean ± standard deviation (SD). Group means were compared with the Mann-Whitney test using SPSS 12.0 K or analysis of variance (ANOVA) followed by Newman-Keuls *post hoc* analysis using GraphPad Prism version 8 (GraphPad, San Diego, CA). Statistical significance was determined when the *P*-value was <0.05.

## Results

### Uremic Toxin Mixtures Mimic the Effects of Uremic Serum on iPSC-ECs

To establish a baseline for studying uremic vasculopathy, we first examined the effects of uremic serum collected from ESRD patients or normal serum from healthy volunteers on iPSC-ECs. Several parameters including cell viability, generation of reactive oxygen species (ROS), and apoptosis were measured following exposure of iPSC-ECs to uremic or normal serum. The blood urea nitrogen and serum creatinine concentrations of uremic serum collected immediately before hemodialysis are summarized with the baseline characteristics of ESRD patients in Table 1. The mean duration of dialysis was 9.8 years and hypertension was present in all ESRD patients.

**Table 1:** Baseline characteristics of normal controls and ESRD patients.

**Characteristics**	**Normal controls (*n* = 5)**	**ESRD patients (*n* = 5)**	***P*-value**
Age, years	32 ± 7.6	49 ± 11.1	0.0317
Male: female	3: 2	3: 2	>0.9999
BMI (kg/m^2^)	23.9 ± 4.43	24.5 ± 6.52	>0.9999
Blood urea nitrogen (mg/dL)	12.48 ± 2.119	51.04 ± 11.32	0.0079
Serum creatinine (mg/dL)	0.62 ± 0.110	10.44 ± 3.480	0.0079
Serum uric acid (mg/dL)	5.24 ± 1.322	7.72 ± 2.100	0.0635
Causes of ESRD, *n* (%)	NA		NA
Diabetes mellitus		1 (20%)	
Hypertension		1 (20%)	
Glomerulonephritis		2 (40%)	
ADPKD		1 (20%)	
Cardiovascular diseases, *n* (%)	NA		NA
Hypertension		5 (100%)	
Pericardial effusion		2 (40%)	
Diastolic dysfunction		1 (20%)	
Aortic dissection		1 (20%)	
Duration of hemodialysis, years	NA	9.8 (2–15)	NA

*Laboratory values are mean ± standard deviation*.

*ADPKD, autosomal dominant polycystic kidney disease; BMI, body mass index; ESRD, end-stage renal disease; NA, not applicable*.

**Figure 1:**
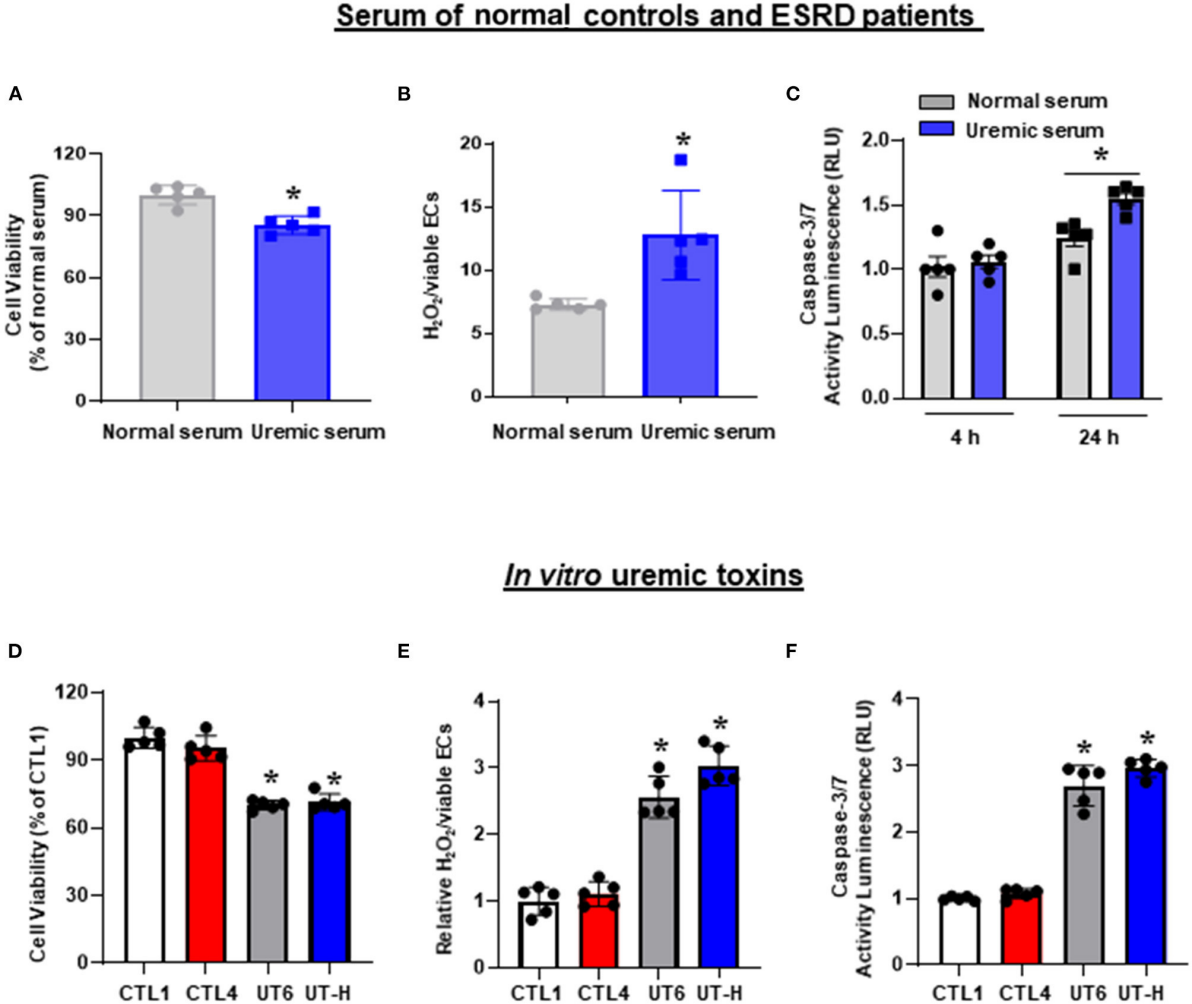
The effects of uremic serum and *in vitro* uremic toxin mixtures on cell viability, ROS, and apoptosis of iPSC-ECs. **(A)** Uremic serum from ESRD patients significantly decreased the cell viability of iPSC-ECs. **(B)** ROS production increased in iPSC-ECs cultured with uremic serum. **(C)** Apoptosis of iPSC-ECs was enhanced by uremic serum at 24 h. **(D)** UT6 and UT-H significantly decreased the cell viability of iPSC-ECs. **(E)** UT6 and UT-H significantly increased ROS production of iPSC-ECs. **(F)** UT6 and UT-H significantly increased apoptosis of iPSC-ECs. *P < 0.05 compared to iPSC-ECs cultured with normal serum or CTL1. *n* = 5 /group. CTL, control; ESRD, end-stage renal disease; iPSC-ECs, induced pluripotent stem cells-derived endothelial cells; ROS, reactive oxygen species; UT6, uremic toxin mixture 6 (containing high concentrations of urea, creatinine, uric acid, indoxyl sulfate, and AGE); UT-H, uremic toxin mixture H (containing high concentrations of urea, creatinine, uric acid, and indoxyl sulfate).

iPSC-ECs treated with uremic serum exhibited a significant decrease in cell viability (Figure 1A) accompanied by a significant increase in ROS generation measured by H_2_O_2_ production (Figure 1B) compared to iPSC-ECs exposed to normal serum. In addition, the degree of apoptosis measured by caspase 3/7 activity in iPSC-ECs treated with uremic serum was not discernible at 4 h compared to those cultured in normal serum but was significantly enhanced by 24 h post-treatment (Figure 1C). Collectively, these results demonstrated that iPSC-ECs exposed to uremic serum collected from ESRD patients exhibited hallmarks of endothelial dysfunction.

Having demonstrated that uremic serum induces endothelial dysfunction in iPSC-ECs, we then sought to identify a minimal *in vitro* mixture of uremic toxins that would elicit similar effects on iPSC-ECs. To achieve this, we tested 25 combinations of substances known to be present in uremic serum (Table 2) based on three parameters: cell viability, ROS production, and apoptotic activity. We first compared control conditions and found that there were no significant differences in cell viability, ROS levels, and caspase 3/7 activity between control (CTL) 1 consisting of media only and CTL4 (media containing physiological levels of urea, creatinine, and uric acid). Subsequent testing revealed that compared with CTL1 and CTL4, exposure of iPSC-ECs in two different combinations of uremic toxin mixture, namely UT6 (containing a high concentration of urea, creatinine, uric acid, indoxyl sulfate, and AGE) and UT-H (containing a high concentration of urea, creatinine, uric acid, and indoxyl sulfate) led to significantly lower cell viability (Figure 1D). Likewise, both ROS production (Figure 1E) and apoptosis (Figure 1F) were significantly elevated in cells exposed to UT6 and UT-H.

**Table 2:** Composition of *in vitro* uremic toxin mixtures.

**Groups**	**Urea, mM (BUN, mg/dL)**	**Creatinine, mM (mg/dL)**	**Uric acid, mM (mg/dL)**	**Indoxyl sulfate (mM)**	**AGE (mg/L)**	**NaOH (μL/mL)**
Control 1 (media only)						
Control 2	5 (14.01)	0.1 (1.13)				
Control 3	5 (14.01)	0.1 (1.13)	0.25 (4.20)			11
Control 4	5 (14.01)	0.1 (1.13)	0.25 (4.20)			
Control 5						16
UA 1			0.5 (8.41)			
UA 2			0.8 (13.45)			
UA 3			1 (16.81)			
Urea	50 (142.86)					
Creatinine		1 (11.30)				
IS			1 (16.81)			
UT 1	50 (142.86)	1 (11.30)				
UT 2	50 (142.86)	1 (11.30)	1 (16.81)			
UT 3	50 (142.86)	1 (11.30)	1 (16.81)	0.5		
UT 4	50 (142.86)	1 (11.30)	1 (16.81)	0.5	1	
UT 5	25 (71.43)	1 (11.30)	1 (16.81)	0.5	10	
UT 6	50 (142.86)	1 (11.30)	1 (16.81)	0.5	10	
UT-A	25 (71.43)	1 (11.30)	0.5 (8.41)			
UT-B	25 (71.43)	1 (11.30)	1 (16.81)			
UT-C	50 (142.86)	1 (11.30)	0.5 (8.41)			
UT-D	50 (142.86)	1 (11.30)	1 (16.81)			
UT-E	25 (71.43)	1 (11.30)	0.8 (13.45)			
UT-F	25 (71.43)	1 (11.30)	0.8 (13.45)	1		
UT-G	50 (142.86)	1 (11.30)	0.8 (13.45)			
UT-H	50 (142.86)	1 (11.30)	0.8 (13.45)	1		

*AGE, advanced glycation end-products*.

### Effects of Uremic Serum and Uremic Toxin Mixtures on the Angiogenetic Ability of iPSC-ECs

**Figure 2:**
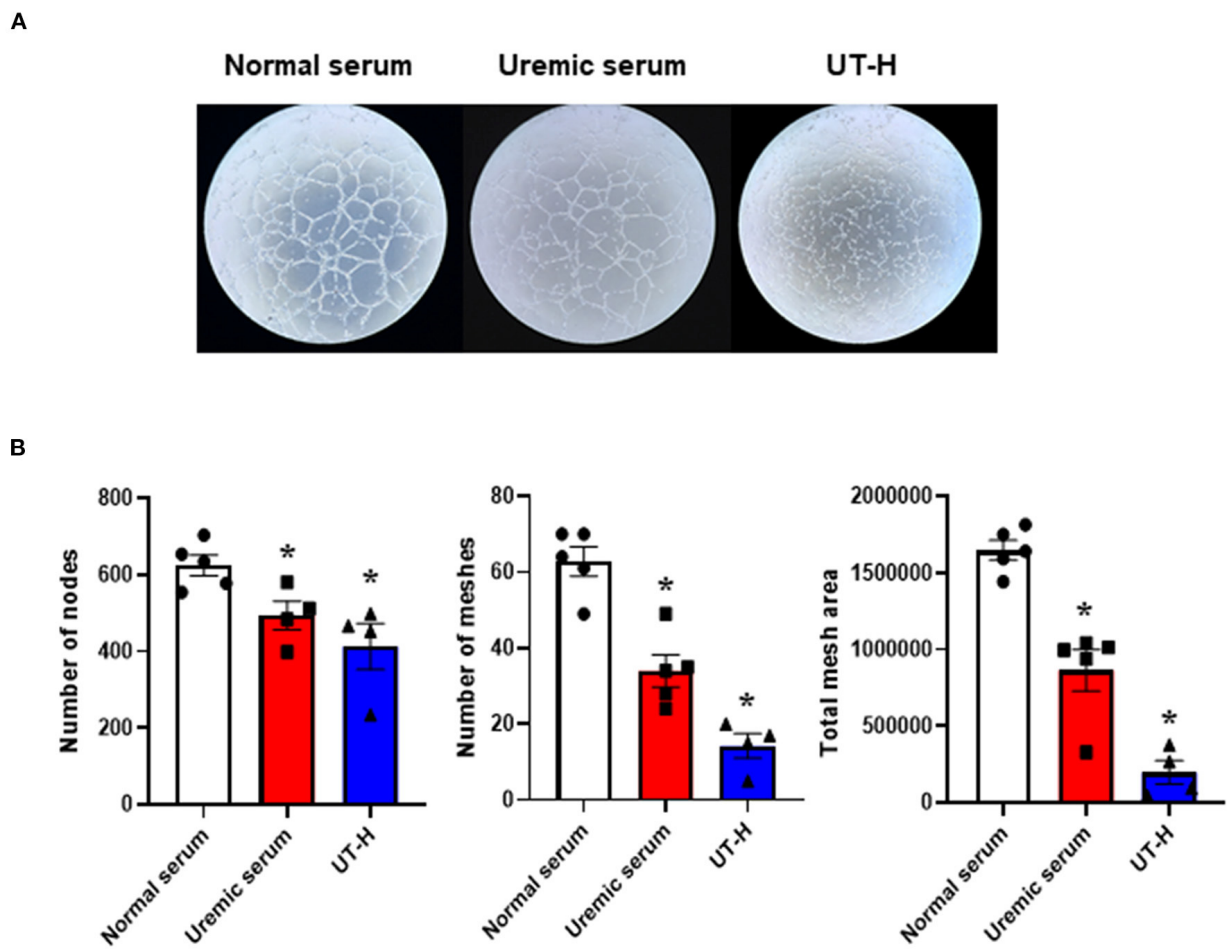
The effects of uremic serum and *in vitro* uremic toxin mixture on vessel formation ability of iPSC-ECs. **(A)** Tube formation of iPSC-ECs was suppressed by uremic serum or UT-H. **(B)** Quantitative data from the tube formation assay. Uremic toxin mixtures exerted comparable effects with uremic serum on tube formation of iPSC-ECs. *P < 0.05 compared to normal serum. *n* = 4–5/group. UT-H, uremic toxin mixture H (containing high concentrations of urea, creatinine, uric acid, and indoxyl sulfate).

**Figure 3:**
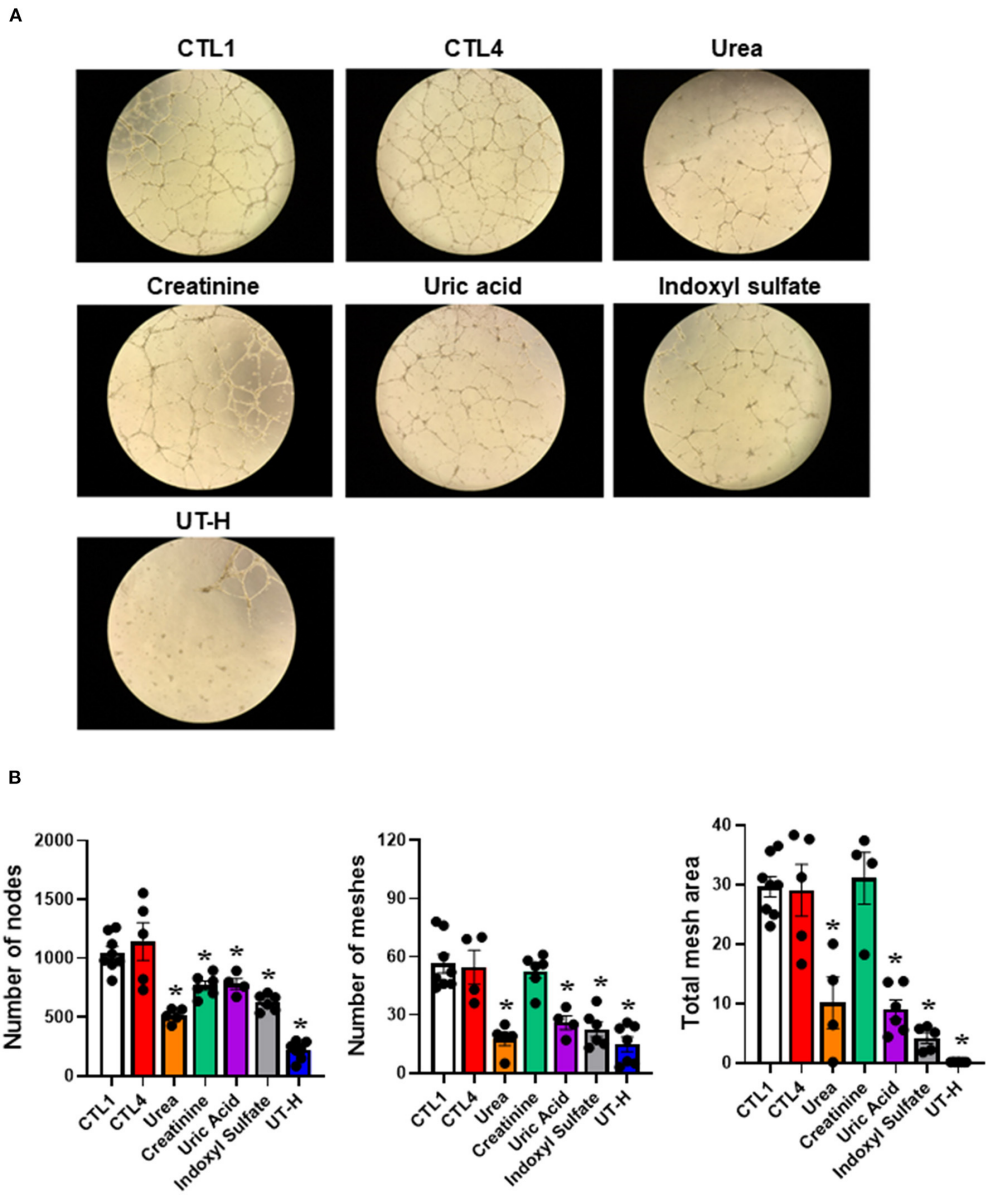
The effects of each uremic toxin on vessel formation ability of iPSC-ECs. **(A)** Tube formation of iPSC-ECs was suppressed by each uremic toxin or UT-H. **(B)** Urea, uric acid, or indoxyl sulfate consistently suppressed tube formation of iPSC-ECs. *P < 0.05 compared to CTL1. *n* = 4–5/group. CTL1, control 1; CTL4, control 4; UT-H, uremic toxin mixture H (containing high concentrations of urea, creatinine, uric acid, and indoxyl sulfate).

We next sought to explore how uremic serum or uremic toxin mixture affect the angiogenic capability of iPSC-ECs. As shown in Figures 2A,B we observed that uremic serum significantly suppressed tube formation of iPSC-ECs determined by the number of nodes and meshes and the total area of meshes compared to normal serum. Similar findings were also revealed in iPSC-ECs supplemented with UT-H mimicking the effect of uremic serum. High concentrations of urea, uric acid, or indoxyl sulfate consistently suppressed tube formation of iPSC-ECs (Figure 3).

### Activation of Intracellular TGF-β Pathway in iPSC-ECs by *in vitro* Uremic Toxin Mixtures and the Different Expression of Inflammatory Cytokines in Uremic Serum

**Figure 4:**
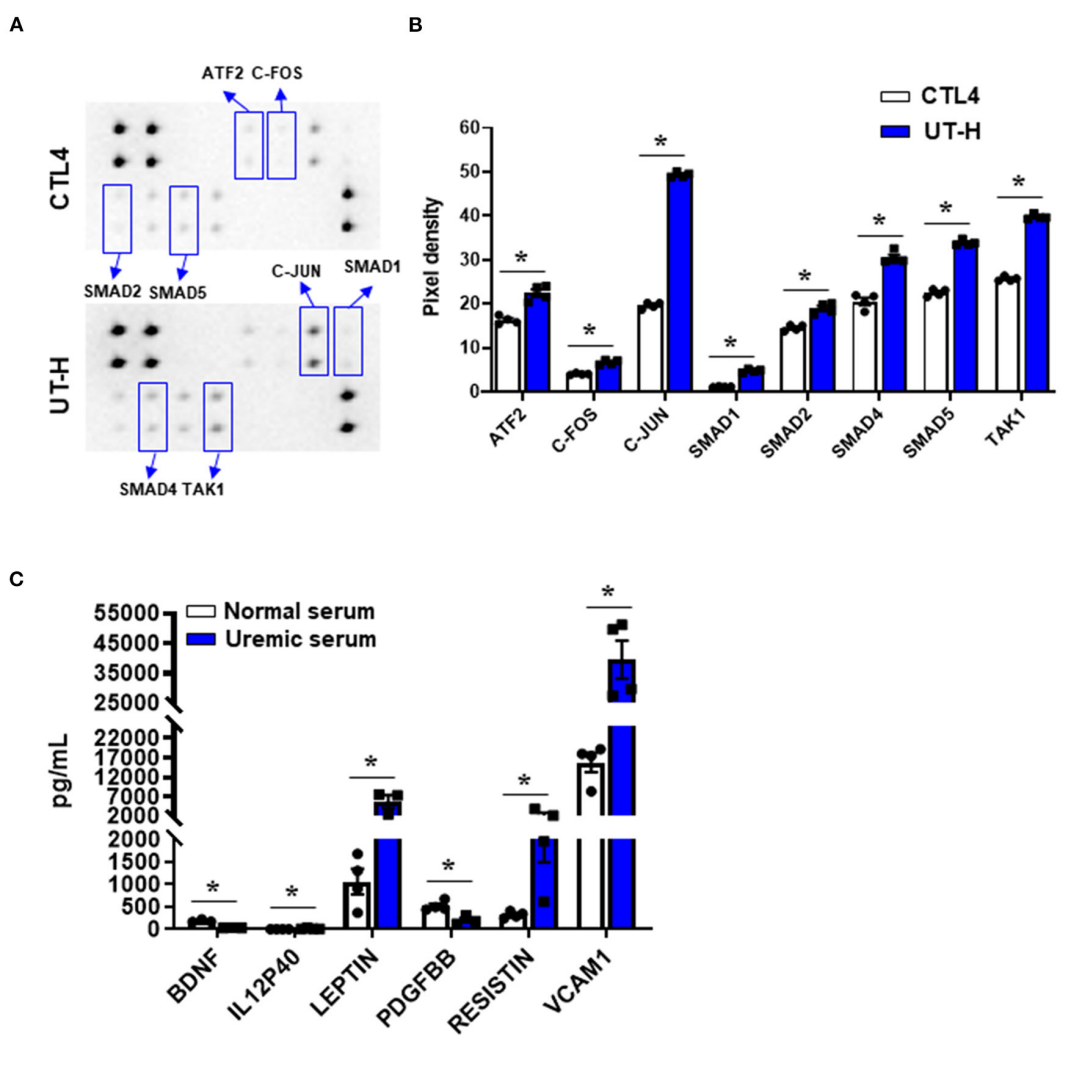
The effects of uremic toxin mixtures on the intracellular TGF-β pathway of iPSC-ECs and inflammatory cytokine levels in uremic serum. **(A)** Duplicated signals of 8 TGF-β pathway phosphorylated proteins. **(B)** Quantitative and statistical analyses of each detected target. *P < 0.05 compared to CTL4. *n* = 4/group. CTL4, control 4 (media containing normal physiologic levels of urea, creatinine, and uric acid); UT-H, uremic toxin mixture H (containing high concentrations of urea, creatinine, uric acid, and indoxyl sulfate). **(C)** Luminex multiplex assay of normal and uremic serum. IL-12P40, leptin, resistin, and VCAM1 were higher in uremic serum than in normal serum. *P < 0.05 compared to normal serum. *n* = 4/group. BDNF, Brain-derived neurotrophic factor; IL-12P40, interleukin-12 P40; PDGF BB, platelet-derived growth factor −BB; VCAM1, vascular cell adhesion molecule 1.

TGF-β has been implicated as a major regulatory cytokine playing important and diverse roles in CKD (Patel and Dressler, 2005). To elucidate the mechanisms underlying the reduction of viability and angiogenesis and the elevation of ROS production and apoptosis in iPSC-ECs treated with uremic toxin mixtures or uremic serum, we first determined whether uremic toxin mixtures induce activation of TGF-β signaling in ECs. Phosphorylation levels of 8 TGF-β pathway proteins were measured in iPSC-ECs after treating cells with either CTL4 or UT-H (Figure 4A). We found that UT-H treatment significantly increased the phosphorylation of all 8 TGF-β pathway proteins, ATF2, C-FOS, C-JUN, SMAD1, SMAD2, SMAD4, SMAD5, and TAK1, compared to CTL4 treatment (Figure 4B), suggesting that the inhibition of TGF-β signaling may protect against uremic toxins.

In addition, to analyze the differences in humoral factors between normal serum and uremic serum, we measured the levels of 62 human inflammatory cytokines in serum from normal controls and ESRD patients (Supplementary Table 1). Of the 62 inflammatory cytokines measured, brain-derived neurotrophic factors (BDNF) and platelet-derived growth factor −BB (PDGF BB) were significantly decreased in uremic serum compared with normal serum, whereas interleukin (IL)-12 p40, leptin, resistin, and vascular cell adhesion molecule 1 (VCAM1) were significantly increased in uremic serum (Figure 4C). In addition, most other cytokines including TGF-β (*P* = 0.057) demonstrated an increasing trend in uremic serum compared to normal serum (Supplementary Table 1).

### Drug Screening Using a Simplified Uremic Vasculopathy Model With iPSC-ECs and Uremic Toxin Mixture

**Figure 5:**
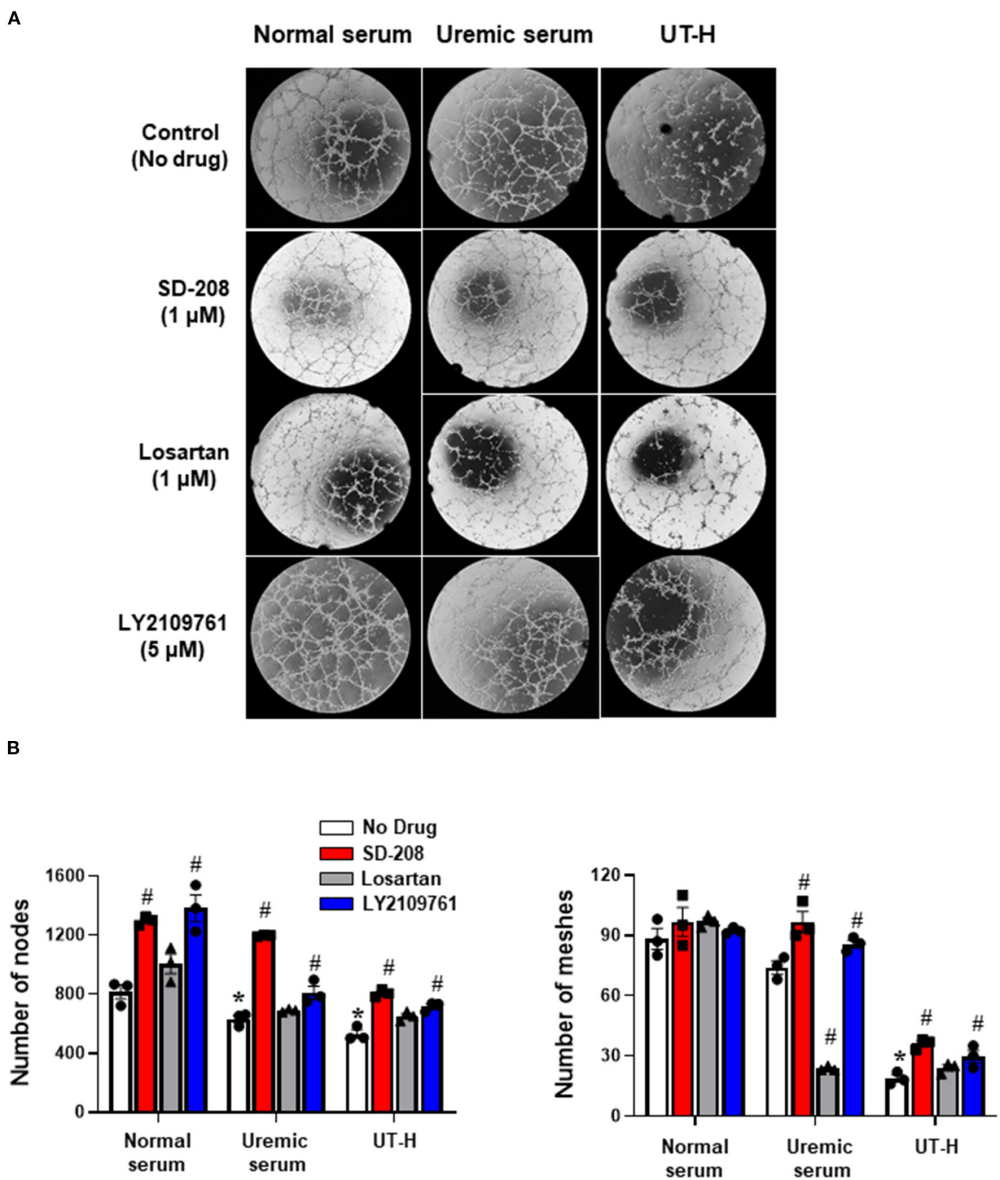
Drug screening using a simplified uremic vasculopathy model. **(A)** The tube formation of iPSC-ECs was significantly suppressed in the uremic serum and UT-H groups. SD-208 and LY2109761 attenuated the adverse effects of uremic serum and UT-H on iPSC-ECs. The formation of capillary-like structures was imaged by phase-contrast microscopy (×20). **(B)** Quantitative data from the tube formation assay. Both SD-208 and LY2109761 showed favorable effects on the tube formation of iPSC-ECs treated with uremic serum or UT-H. Losartan also exerted favorable effects on iPSC-ECs treated with the UT-H but not significant. *P < 0.05 compared to normal serum group with no drug. ^**#**^*P* < 0.05 compared to no drug within the same group. *n* = 3/group. UT-H, uremic toxin mixture H (containing high concentrations of urea, creatinine, uric acid, and indoxyl sulfate).

We next sought to test whether the inhibition of TGF-β signaling reduces uremic toxin-induced impairment of endothelial function. iPSC-ECs were incubated with three drugs targeting the TGF-β pathway, namely SD-208 (TGF-β type 1 receptor inhibitor), losartan (TGF-β signaling blocker), and LY2109761 (dual inhibitor of TGF-β type 1 and 2 receptor inhibitor) before TGF-β stimulation *via* treatment with uremic serum or UT-H. Treatment with SD-208 and LY2109761 significantly restored the formation of tubes in iPSC-ECs incubated with uremic serum and UT-H (Figure 5), which suggests that the inhibition of TGF-β signaling is a protective mechanism against uremic vasculopathy.

**Figure 6:**
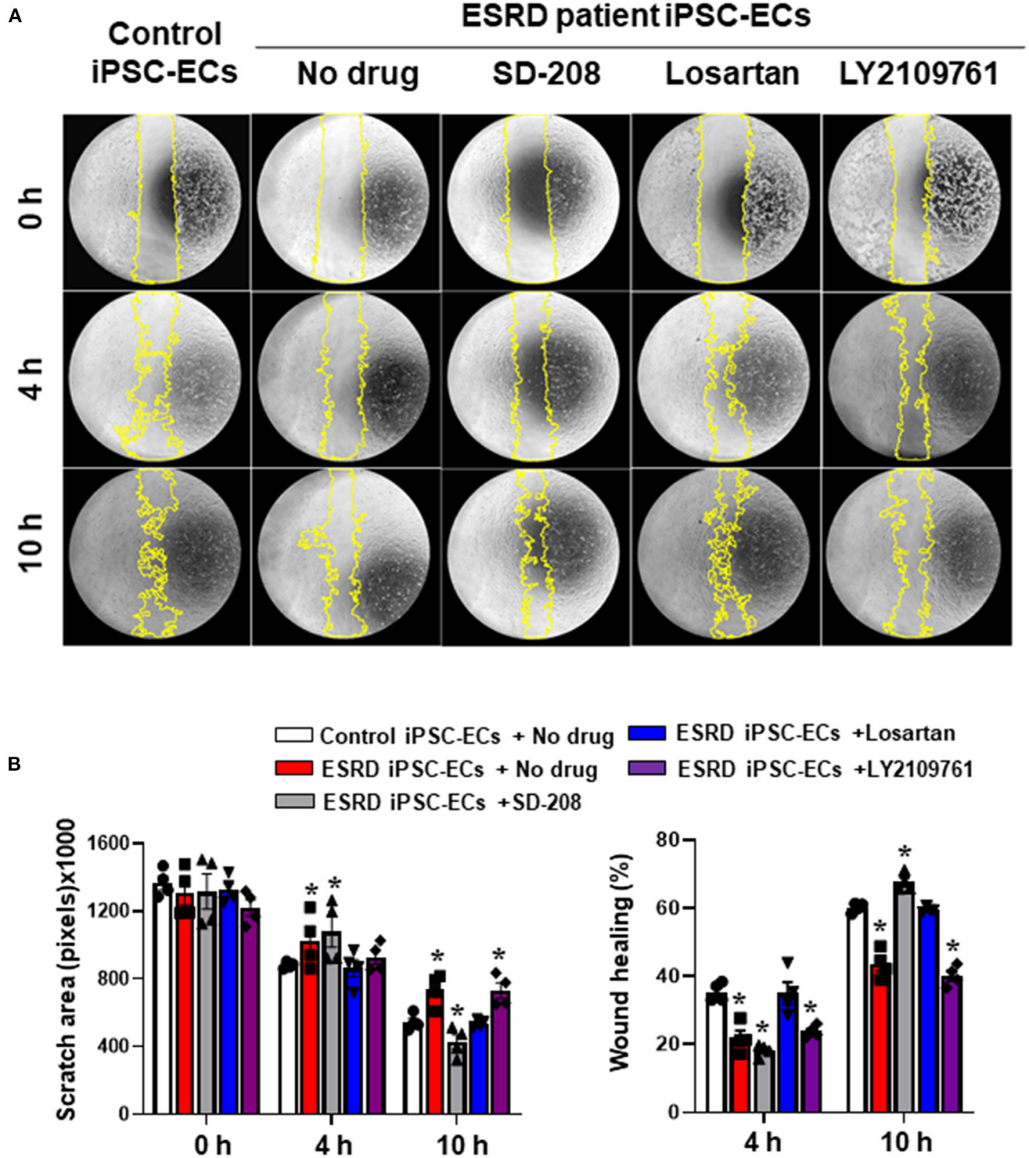
Preliminary drug screening using ESRD patient-specific iPSC-ECs. **(A)** The degree of wound healing evaluated by a migration assay was significantly impaired in the ESRD patient-specific iPSC-ECs compared to the control iPSC-ECs. **(B)** Quantitative data from the migration assay. Losartan and SD-208 facilitated wound healing of the ESRD patient-specific iPSC-ECs. *P < 0.05 compared to the control iPSC-ECs with no drug. Control iPSC-ECs, iPSC-ECs from a normal control; ESRD, end-stage renal disease; ESRD iPSC-ECs, iPSC-ECs from an ESRD patient receiving hemodialysis.

To further validate our *in vitro* model as a drug screening model, we generated iPSCs from an ESRD patient and compared the newly generated ESRD patient-specific iPSC line against normal control iPSCs to determine whether patient-specific iPSC-ECs can potentially recapitulate susceptibility against uremic vasculopathy. ESRD iPSCs exhibited typical expression of pluripotency markers (Supplementary Figure 1A). The differentiation efficiency and expression of endothelial markers of ESRD patient-specific iPSC-ECs were also comparable to those of normal control iPSC-ECs (Supplementary Figure 1B). The functional integrity of normal control and ESRD patient-specific iPSC-ECs was then compared by tube formation assay and migration assay. ESRD patient-specific iPSC-ECs showed impaired tube formation compared to normal control iPSC-ECs (Supplementary Figure 2). In the migration assay, the degree of wound healing was significantly lower in ESRD patient-specific iPSC-ECs (Figure 6A) compared to control iPSC-ECs. Interestingly, losartan and SD-208 significantly mitigated the impairment of wound healing in ESRD patient-specific iPSC-ECs (Figure 6B) demonstrating that patient-specific iPSC-ECs represent a model that may be used for personalized drug screening in the future.

### Gene and Functional Enrichment Analysis With the Addition of Uremic Toxin Mixture

**Figure 7:**
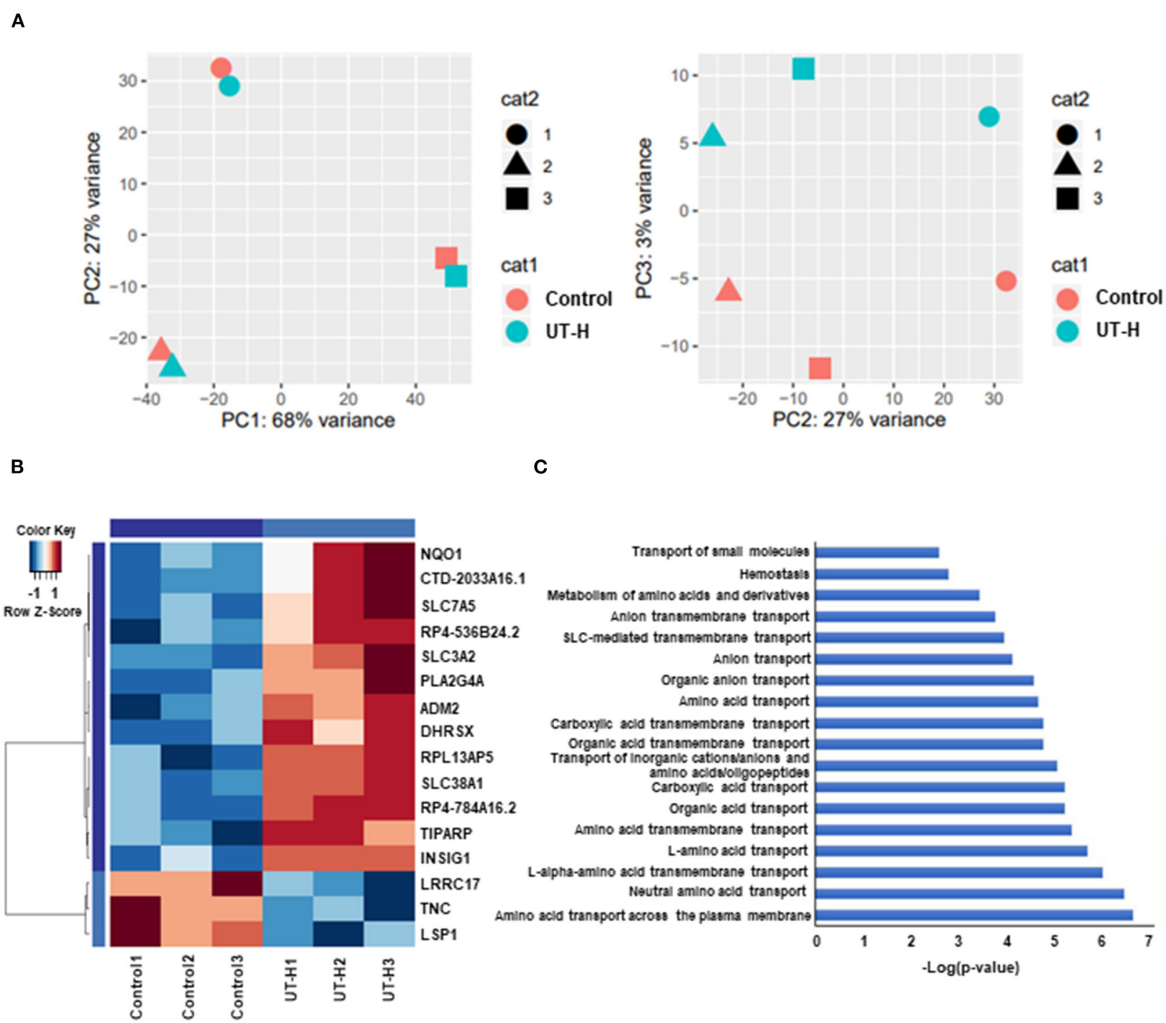
Whole transcriptome analysis of treatment with uremic toxin mixture. **(A)** Principal component analysis (PCA) of gene expression profiles from indicated samples, shown as a scatter plot of PC1 vs. PC2 and PC2 vs. PC3. **(B)** Hierarchical clustering of differentially expressed genes (DEGs) between the UT-H treated group and control group. **(C)** Gene ontology (GO) pathway analysis of DEGs. UT-H, uremic toxin mixture (containing high concentrations of urea, creatinine, uric acid, and indoxyl sulfate)

Finally, to better understand the effects of UT-H on the transcriptome profiles of iPSC-ECs (GEO accession number: RNA-seq num GSE155969), RNA-sequencing was conducted. Principal component analysis (PCA) was first conducted to examine the main source of variation in the data. PC1 reflected a closer correlation in iPSC-ECs from the same patient whereas PC3 identified distinct clusters corresponding to UT-H treated control samples (Figure 7A). Hierarchical clustering analysis (Figure 7B) of differentially expressed genes (DEGs) revealed different gene expression patterns after exposure to UT-H compared with control groups. In addition, a functional enrichment analysis was conducted to reveal the biological process and molecular function involved in a simplified *in vitro* uremic vasculopathy model. The most preserved expression profiles between control and UT-H treated samples were related to the amino acid transport across the plasma membrane, neutral amino acid transport, L-alpha-amino acid transmembrane transport, L-amino acid transport, as well as amino acid transmembrane transport (Figure 7C).

## Discussion

Complex and diverse systemic dysfunction involving nearly all major organs is common in ESRD patients, with cardiovascular complications being the leading cause of death (Tonelli et al., 2006; Stenvinkel, 2010). Endothelial dysfunction is not only an initiating pathogenic factor but also the cornerstone in the progression of cardiovascular complications in CKD (Aznar-Salatti et al., 1995; Merino et al., 2008; Kramann et al., 2012). Uremic vasculopathy is an accelerated atherosclerotic disease frequently accompanied by severe vascular calcification under the uremic micromilieu of CKD patients (Martin-Rodriguez et al., 2015; Guo et al., 2017). Although a few uremic toxins are known to cause endothelial damage (Glorieux and Vanholder, 2011), the types or concentration of uremic toxins triggering uremic vasculopathy is not fully understood. In this study, we have demonstrated that an *in vitro* uremic milieu comprising of urea, creatinine, uric acid, and indoxyl sulfate leads to impairment of iPSC-EC functional integrity. Importantly, these effects were comparable to the effects of uremic serum collected from ESRD patients but are more reproducible and consistent. We have also shown that this simplified uremic vasculopathy model is potentially amenable to drug screening by revealing that TGF-β signaling inhibitors are capable of partially reversing the defects seen in iPSC-ECs exposed to uremic toxin mixtures. Lastly, we have shown that patient-specific iPSC-ECs derived from an ESRD patient exhibited a basal dysfunctional state compared to normal control iPSC-ECs, which can also be rescued by TGF- β inhibitors.

Cell culture models are often used for simulating uremic vasculopathy in humans. For instance, human umbilical vein endothelial cells (HUVECs) are commonly exposed to 10–20% diluted serum of CKD patients (Chitalia et al., 2011; Lanza et al., 2015). HUVECs have been most frequently used in cell culture models of uremic vasculopathy. However, these cells often do not reflect disease susceptibility because of their unique characteristics mimicking stem cells such as hematopoietic support (Yamaguchi et al., 1998; Bal et al., 2012, 2013). iPSC-ECs in our study may be superior to HUVECs in reflecting genetic susceptibility to uremic vasculopathy. It remains a challenge to discern the effects of each uremic toxin *per se* on ECs using the serum of patients since serum usually contains a complex mixture of confounders such as cytokines and parathyroid hormones (Castillo-Rodríguez et al., 2017). In addition, differences in the concentration of each uremic toxin between individuals as well as temporal difference within the same patient also limit the usefulness of serum as a research tool. Moreover, there is also the ethical issue of acquiring large volumes of serum since most CKD patients are anemic. Based on these limitations, in this study, we investigated the effects of a simplified mixture of several well-known uremic toxins on iPSC-ECs, which were chosen by clinical criteria used to evaluate the degree of uremia as well as the pathogenic roles reported in previous studies.

Urea and creatinine are typical uremic toxins, most commonly used to evaluate CKD patients in clinics (Lau and Vaziri, 2017). Urea substantially contribute to the progression atherosclerosis by inducing endothelial dysfunction, endothelial progenitor cells senescence, and apoptosis of vascular smooth muscle cells (Giardino et al., 2017). Uric acid is known as an important uremic toxin contributing to CKD progression (Gustafsson and Unwin, 2013; Tsai et al., 2017) and increasing risk of cardiovascular mortality in CKD patients (Luo et al., 2019). Urea, creatinine, and uric acid are widely used as essential parameters to evaluate CKD patients in nephrology clinics since these toxins are easily measurable under internationally standardized protocols. Indoxyl sulfate and AGE were reported to increase cardiovascular risk in CKD (Wang et al., 2005; Ito et al., 2013; Barisione et al., 2015). Indoxyl sulfate, the most representative gut-derived uremic toxin, shows endothelial toxicity and subsequently plays a substantial role in the progression of atherosclerosis by inhibiting endothelial proliferation and repair (Hung et al., 2017; Lano et al., 2020). Indoxyl sulfate also impaired the functional ability of human mesenchymal stem cells (Wang et al., 2016). Our data showed that a mixture of high concentrations of urea, creatinine, uric acid, and indoxyl sulfate can consistently mimic the detrimental effects of uremic serum on iPSC-ECs. Treatment with high concentrations of urea, uric acid, or indoxyl sulfate also significantly impaired tube formation of iPSC-ECs, supporting the vascular toxicity of these uremic toxins reported in previous studies. The physiologic concentrations of urea, creatinine, and uric acid on the other hand did not cause adverse effects on iPSC-ECs, supporting our hypothesis that a simplified mixture of highly concentrated urea, creatinine, uric acid, and indoxyl sulfate can exert adverse effects on iPSC-ECs mimicking uremic serum.

Using this *in vitro* uremic vasculopathy model, we successfully elucidated that impaired TGF-β signaling is a potential pathogenic mechanism leading to impaired endothelial function in iPSC-ECs. This is in accordance with previous reports demonstrating that perturbed TGF-β signaling induces endothelial-mesenchymal transition enhancing fibrosis and non-hereditary disorders such as atherosclerosis and cardiac fibrosis (Pardali et al., 2017; Goumans and Ten Dijke, 2018). Mutations in the TGF-β superfamily have also been shown to disrupt endothelial patterning and induce arteriovenous malformations (Sugden and Siekmann, 2018). We also tested the feasibility of this simplified uremic vasculopathy model of iPSC-ECs for the screening of potential therapeutics. Losartan and TGF-β inhibitors were chosen for drug screening based on the previous studies reporting cardiovascular protective effects of angiotensin receptor blockers in CKD (Kuriyama et al., 2006; Verbeke et al., 2014; Kim-Mitsuyama et al., 2018) and our data showing the activation of intracellular TGF-β pathway in iPSC-ECs by *in vitro* uremic toxin mixtures. Both drugs attenuated the adverse effects of uremic toxins on normal control iPSC-ECs which is in line with a previous study reporting that the inhibition of TGF-β signaling leads to the improved vascular network formation by iPSC-ECs (Kurokawa et al., 2017). As a further extension of our work, we also demonstrated that ESRD patient-specific iPSC-ECs revealed impaired wound healing ability compared to normal control iPSC-ECs demonstrating patient-specific iPSC-ECs recapitulate clinical susceptibility to a certain extent. Accordingly, the degree of wound healing was improved by losartan and SD-208 in ESRD iPSC-ECs. These results support the promising role of a simplified uremic vasculopathy model of iPSC-ECs as a novel drug screening tool reflecting disease susceptibility to uremic toxins.

Our study has a few limitations that should be addressed by future studies. First, more quantitative methodological approaches including functional genomics are required for improving the clinical usefulness of this model as a drug screening system. Although several quantitative or semiquantitative methods were used for analyzing phenotypic changes of iPSC-ECs in this study, more sensitive, as well as quantitative methods, are required for high throughput drug screening. Second, our study did not implement a standard method for evaluating the functional integrity of iPSC-ECs and there were some variations in evaluating the adverse effects of uremic toxins depending on the methods. A standardization process will be required for applying this approach in clinical practice. Third, we did not investigate aging effects on iPSC-ECs. Since the incidence of CKD and the risk of CKD progression to ESRD usually increase with age, further studies investigating differences in iPSC-ECs from young or old CKD patients would be required to improve clinical feasibility of this model.

## Conclusion

Collectively, this study reports a novel platform of using iPSC-ECs to study uremic vasculopathy induced by a simplified uremic toxin mixture that can be applied as a potential drug screening system. Our data demonstrate that a simplified uremic toxin mixture can simulate the uremic micromilieu more reproducibly and consistently than uremic serum from ESRD patients. The deleterious effects of uremic toxins on iPSC-ECs were partly attenuated by an angiotensin-receptor blocker and TGF-β inhibitors supporting the potential usefulness of this model as a drug screening system. This new model of uremic vasculopathy represents a novel way to investigate the pathophysiology behind CKD and can be used to screen both conventional and new drugs.

## Data Availability Statement

The datasets generated for this study can be found in online repositories. The names of the repository/repositories and accession number(s) can be found at: NCBI GEO [accession: GSE155969].

## Ethics Statement

The studies involving human participants were reviewed and approved by the Institutional Review Board of the Samsung Medical Center. The patients/participants provided their written informed consent to participate in this study.

## Author Contributions

HRJ, HJC, WHL, and Y-GK wrote the first draft of the manuscript. HRJ, HJC, WH, S-GO, WHL, and Y-GK supervised the experiments. HRJ, YZ, N-YS, KL, JJ, JEL, and HL analyzed the data. All authors contributed to the article and approved the submitted version.

## Conflict of Interest

The authors declare that the research was conducted in the absence of any commercial or financial relationships that could be construed as a potential conflict of interest.

## Supplementary Material

Supplemental Figure 1 iPSCs and iPSC-ECs of a normal control and an ESRD patient. **(A)** ESRD patient-specific iPSCs showed comparable expression of iPSC pluripotency markers, such as Nanog, COT4, TRA-1-60, and SSEA-4, compared to the normal control iPSCs. **(B)** ESRD patient-specific iPSC-ECs had a similar expression of endothelial markers including VWF, CD144 (VE-Cadherin), and CD31 compared to control iPSC-ECs. DAPI, 4′,6-diamidino-2-phenylindole; ESRD, end-stage renal disease; OCT4, octamer-binding transcription factor 4; SSEA4, stage-specific embryonic antigen 4; TRA-1-60, podocalyxin; VE-Cadherin, vascular endothelial cadherin; VWF, von-Willebrand factor.

Supplementary Figure 2 Tube formation of iPSC-ECs from a normal control and an ESRD patient. **(A)** Vessel structure formation ability of iPSC-ECs from a normal control and an ESRD patient was compared with tube formation. **(B)** Tube formation was significantly impaired in ESRD patient-specific iPSC-ECs compared to normal control iPSC-ECs. ^*^*P* < 0.05 compared to the control iPSC-ECs. ESRD, end-stage renal disease.
