# Supplementary material for: Modeling Uremic Vasculopathy With Induced Pluripotent Stem Cell-Derived Endothelial Cells as a Drug Screening System
Source: Front Cell Dev Biol. 2021 Jan 12;8:618796. doi: 10.3389/fcell.2020.618796 (PMC7835337; doi:10.3389/fcell.2020.618796)
Supplement: Supplementary file 1 [file Table_1.docx]

**Supplemental Table 1.** Mean cytokine levels in normal and uremic serum.

| **Target** | **Normal serum (n=4)**  **pg/mL** | **Uremic serum (n=4)**  **pg/mL** | ***P* value** |
| --- | --- | --- | --- |
| **BDNF** | 188.8 ± 31.29 | 34.49 ± 2.1 | 0.001* |
| **CD40L** | 55.49 ± 22.97 | 194.43 ± 277.05 | 0.486 |
| **EGF** | 26.69 ± 9.84 | 18.3 ± 13.61 | 0.358 |
| **ENA78** | 91.30 ± 65.46 | 213.1 ± 260.97 | 0.477 |
| **EOTAXIN** | 11.77 ± 4.35 | 27.7 ± 30.71 | 0.486 |
| **FASL** | Undetectable | Undetectable | - |
| **FGFB** | Undetectable | Undetectable | - |
| **GCSF** | 17.50 ± 3.49 | 1534.62 ± 3027.65 | 0.486 |
| **GMCSF** | 0.86 ± 0.80 | 82.90 ± 118.41 | 0.421 |
| **GROA** | Undetectable | Undetectable | - |
| **HGF** | 113.46 ± 12.14 | 473.60 ± 349.80 | 0.086 |
| **ICAM1** | 2677.66 ± 2417.57 | 957.60 ± 987.84 | 0.486 |
| **IFNA** | Undetectable | Undetectable | - |
| **IFNB** | 41.13 ± 30.21 | 387.80 ± 729.31 | 0.886 |
| **IFNG** | Undetectable | Undetectable | - |
| **IL10** | Undetectable | Undetectable | - |
| **IL12P40** | 3.46 ± 0.51 | 15.77 ± 12.23 | 0.029* |
| **IL12P70** | 1.04 ± 0.28 | 13.45 ± 25.02 | 0.886 |
| **IL13** | Undetectable | Undetectable | - |
| **IL15** | Undetectable | Undetectable | - |
| **IL17A** | 10.47 ± 3.15 | 41.39 ± 59.1 | 0.200 |
| **IL17F** | 8.07 ± 4.58 | 821.66 ± 1614.56 | 0.486 |
| **IL18** | 7.50 ± 2.25 | 68.09 ± 116.16 | 0.343 |
| **IL1A** | Undetectable | 80.93 ± 105.97 | - |
| **IL1B** | 0.46 ± 0.09 | 6.70 ± 12.32 | 0.886 |
| **IL1RA** | Undetectable | Undetectable | - |
| **IL2** | 21.46 ± 2.34 | 59.35 ± 85.02 | 0.343 |
| **IL21** | Undetectable | 152.87 ± 210.39 | - |
| **IL22** | Undetectable | Undetectable | - |
| **IL23** | Undetectable | Undetectable | - |
| **IL27** | Undetectable | Undetectable | - |
| **IL31** | Undetectable | Undetectable | - |
| **IL4** | 22.33 ± 9.57 | 148.39 ± 275.74 | 0.686 |
| **IL5** | Undetectable | Undetectable | - |
| **IL6** | Undetectable | Undetectable | - |
| **IL7** | 2.38 ± 0.88 | 6.45 ± 11.02 | 0.343 |
| **IL8** | Undetectable | Undetectable | - |
| **IL9** | Undetectable | Undetectable | - |
| **IP10** | 4.64 ± 3.30 | 110.31 ± 144.55 | 0.333 |
| **Supplemental Table 1. (continued)** | | | |
| **Target** | **Normal serum (n=4)**  **pg/ml** | **Uremic serum (n=4)**  **pg/ml** | ***P* value** |
| **LEPTIN** | 1063.30 ± 563.72 | 5799.02 ± 2886.22 | 0.021* |
| **LIF** | 1.22 ± 0.31 | 28.53 ± 54.11 | 0.686 |
| **MCP1** | 9.60 ± 4.47 | 42.31 ± 59.66 | 0.486 |
| **MCP3** | 28.05 ± 9.90 | 189.32 ± 337.98 | 0.886 |
| **MCSF** | Undetectable | Undetectable | - |
| **MIG** | 72.48 ± 61.25 | 622.54 ± 728.66 | 0.181 |
| **MIP1A** | Undetectable | 560.60 ± 649.05 | - |
| **MIP1B** | 6.79 ± 3.08 | 172.44 ± 300.95 | 0.486 |
| **NGF** | Undetectable | Undetectable | - |
| **PAI1** | 5988.35 ± 1971.09 | 5445.31 ± 1338.20 | 0.665 |
| **PDGFBB** | 518.19 ± 107.25 | 206.71 ± 93.16 | 0.01* |
| **RANTES** | 25.00 ± 1.97 | 28.82 ± 15.61 | 0.644 |
| **RESISTIN** | 323.53 ± 70.33 | 2161.44 ± 1341.09 | 0.029* |
| **SCF** | 1.83 ± 1.27 | 26.85 ± 40.24 | 0.057 |
| **SDF1A** | 219.16 ± 40.50 | 15505.43 ± 24857.54 | 0.057 |
| **TGFA** | 0.78 ± 0.32 | 4.45 ± 8.19 | 0.686 |
| **TGFB** | 4.80 ± 3.2 | 519.78 ± 1011.48 | 0.057 |
| **TNFA** | 29.98 ± 0.78 | 56.44 ± 54.74 | 0.343 |
| **TNFB** | Undetectable | Undetectable | - |
| **TRAIL** | 23.78 ± 6.48 | 2247.7 ± 3610.4 | 0.057 |
| **VCAM1** | 15765.96 ± 4976.13 | 39566.81 ± 12624.21 | 0.029* |
| **VEGF** | 29.28 ± 35.27 | 1361.08 ± 2347.66 | 0.114 |
| **VEGFD** | Undetectable | 38.13 ± 32.80 | - |

Data are presented as mean ± SD. A student’s unpaired t-test (two-tailed) was performed to compare the cytokines between normal serum and uremic serum. Unadjusted *P* values are shown, and an asterisk is noted when comparisons were significant.
