# Supplementary figures and images for: Modeling Uremic Vasculopathy With Induced Pluripotent Stem Cell-Derived Endothelial Cells as a Drug Screening System
Source: Front Cell Dev Biol. 2021 Jan 12;8:618796. doi: 10.3389/fcell.2020.618796 (PMC7835337; doi:10.3389/fcell.2020.618796)

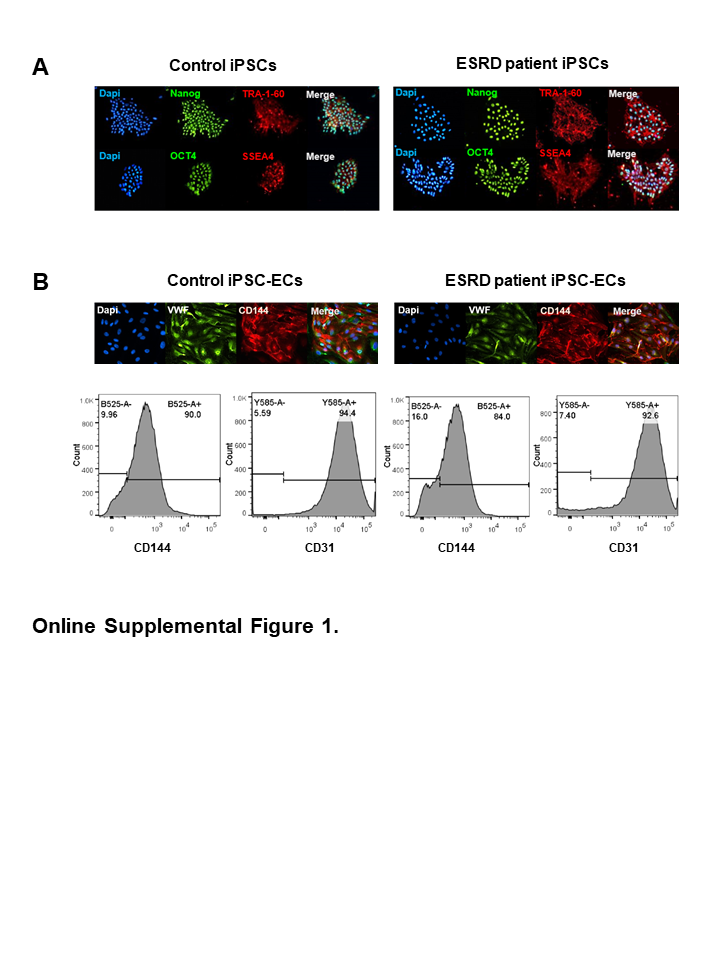

Supplement: Supplemental Figure 1 — iPSCs and iPSC-ECs of a normal control and an ESRD patient. (A) ESRD patient-specific iPSCs showed comparable expression of iPSC pluripotency markers, such as Nanog, COT4, TRA-1-60, and SSEA-4, compared to the normal control iPSCs. (B) ESRD patient-specific iPSC-ECs had a similar expression of endothelial markers including VWF, CD144 (VE-Cadherin), and CD31 compared to control iPSC-ECs. DAPI, 4′,6-diamidino-2-phenylindole; ESRD, end-stage renal disease; OCT4, octamer-binding transcription factor 4; SSEA4, stage-specific embryonic antigen 4; TRA-1-60, podocalyxin; VE-Cadherin, vascular endothelial cadherin; VWF, von-Willebrand factor. [file Image_1.TIF]

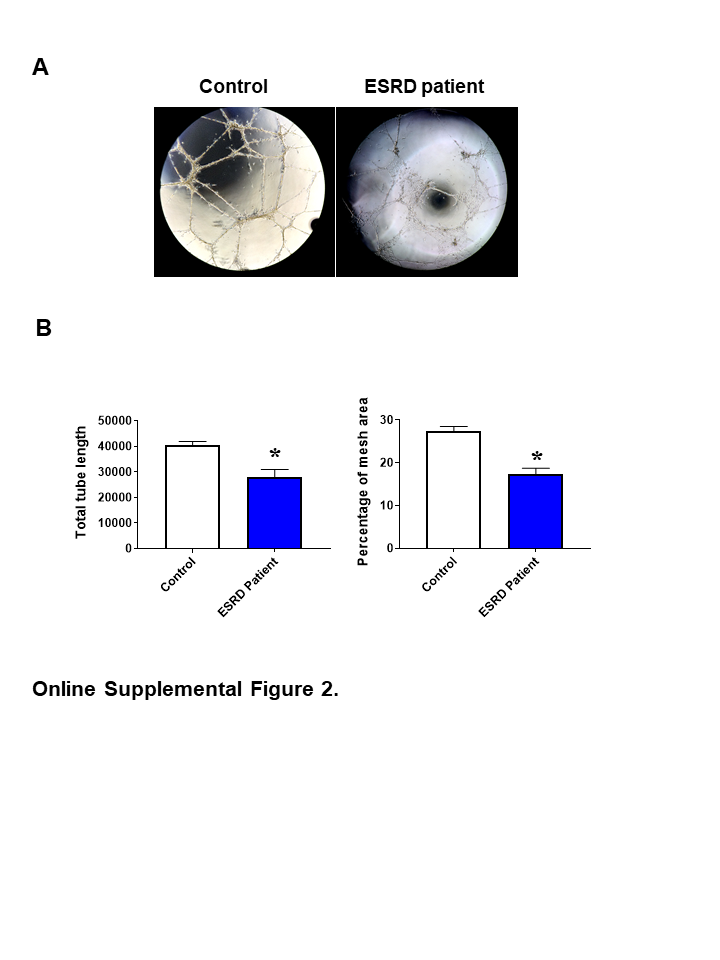

Supplement: Supplementary Figure 2 — Tube formation of iPSC-ECs from a normal control and an ESRD patient. (A) Vessel structure formation ability of iPSC-ECs from a normal control and an ESRD patient was compared with tube formation. (B) Tube formation was significantly impaired in ESRD patient-specific iPSC-ECs compared to normal control iPSC-ECs. *P < 0.05 compared to the control iPSC-ECs. ESRD, end-stage renal disease. [file Image_2.TIF]
